# Serial-position effects in preference construction: a sensitivity analysis of the pairwise-competition model

**DOI:** 10.3389/fpsyg.2014.00902

**Published:** 2014-08-22

**Authors:** Emina Canic, Thorsten Pachur

**Affiliations:** ^1^Max Planck Institute for Human DevelopmentBerlin, Germany; ^2^Department of Psychology, University of WarwickCoventry, UK

**Keywords:** primacy and recency effects, serial-position effects, inertia, sequential evaluation, preference construction

## Abstract

People have a stronger preference for options encountered earlier or later in a sequence than for options in the middle of the sequence. To account for these primacy and recency effects, Mantonakis et al. (2009) sketched a sequential updating mechanism, the *pairwise-competition model*. We propose a formal instantiation of the model and, using computer simulations, examine how the sizes of the predicted primacy and recency effects are affected by (a) variability in the quality of the options; (b) the number of options presented (sequence length); (c) the level of choice inertia (i.e., the tendency to stick with the current favorite); and (d) whether choice inertia dynamically increases over the sequence. We find that recency effects are reduced and primacy effects are increased with variability in quality as compared to without, and that this holds regardless of sequence length. A sizeable primacy effect occurs only with relatively short sequences or rather high levels of choice inertia. Dynamic inertia increases primacy effects and reduces recency effects, and the impact increases with higher inertia levels. We relate these results to empirical findings and derive novel predictions from the model.

A prominent feature of experience is that it unfolds serially, at least to our minds. Due to constraints in attention, we process events and objects one after the other (Simon, 1979). Does this sequentiality of experience shape the decisions we make? It is well known that the way preferences are constructed can be influenced by numerous contextual factors, such as the framing of outcomes as gains or losses, whether a preference is elicited by choice or by evaluation, the emotional content of the task, or the similarity of the available options (Tversky and Kahneman, 1981; Huber et al., 1982; Slovic, 1995; Pachur et al., 2014; for an overview, see Lichtenstein and Slovic, 2006). Moreover, how people search for attribute information can impact their preferences (Weber and Johnson, 2006; Pachur and Scheibehenne, 2012).

Given the constructive nature of preference, it is not surprising that the serial position in which an option is presented appears to impact how it is evaluated. For instance, Miller and Krosnick (1998) analyzed election races and found a *primacy effect*, defined as a tendency to favor the first option in a sequence over options in the middle: candidates listed first received more votes than other candidates. In addition, Bruine de Bruin (2005) obtained evidence for *recency effects*, defined as a tendency to favor the last option in a sequence over options in the middle: contestants in song competitions received higher ratings the later they appeared in the sequence. Page and Page (2010) found both types of serial position effects when analyzing song contest series, with the recency effect being more pronounced than the primacy effect.

Mantonakis et al. (2009) examined variables moderating primacy and recency effects in choice, such as expertise and the number of options presented (i.e., sequence length). They presented participants with either two, three, four, or five wine samples. After participants had tasted all samples they indicated their favorite. Although, unbeknown to participants, each sample was poured from the same bottle, there was a tendency to prefer the wine presented first. In longer sequences, moreover, highly knowledgeable participants (as objectively assessed via a questionnaire) also indicated an increased preference for the sample presented last. Participants with little knowledge about wines did not show a recency effect.

Mantonakis et al. (2009) sketched a process model—which we term the *pairwise-competition model* (PCM)—to account for the recency and primacy effects in preference. In a nutshell, the model assumes that when encountering options sequentially, the decision maker conducts a repeated pairwise comparison process in which each new option is compared with the current favorite (see below for details).

Our goal in this article is to propose a formal implementation of the PCM that accommodates all of the components proposed verbally by Mantonakis et al. (2009), and to systematically test the extent to which the model predicts serial-position effects under various conditions. First, whereas Mantonakis et al. (2009) examined the condition in which all options are of equal quality, we investigate how introducing quality variability affects the size of the primacy and recency effects predicted by the PCM. Second, as expertise was shown to affect the occurrence of serial-position effects, we examine the impact of different levels of *choice inertia* (i.e., the tendency to stick with the current favorite), which may be linked to expertise, on the model’s predictions. Third, we formalize and test Mantonakis et al.’s (2009) proposal that choice inertia might become stronger over the course of the sequence. Fourth, because the authors found that the number of options in the sequence can influence the degree to which serial-position effects emerge, we consider the potentially moderating role of sequence length.

## THE PAIRWISE-COMPETITION MODEL

The PCM combines two mechanisms that have previously been proposed to underlie serial-position effects in preference construction: repeated pairwise comparison (Houston et al., 1989; Bruine de Bruin and Keren, 2003) and a bias for the initially presented option (e.g., Carney and Banaji, 2012). Specifically, the model assumes that a decision maker encountering options sequentially per default initially prefers the first option encountered. The second option is then compared with the first one, and one of the two is chosen. The third option is then compared with the “winner” of the previous comparison, and so on. This sampling and updating process based on repeated pairwise comparisons continues until the last option in the sequence is reached and the final winner has been determined.

According to Mantonakis et al. (2009), the PCM’s “first-is-best bias accounts for the consistent primacy effect” (p. 1311). The assumed pairwise-competition process, by contrast, can produce a recency effect, “especially in the case of longer sequences, in which an early option has to ‘beat’ more options to become the overall favorite” (p. 1310). A recency effect should be reduced, however, if the decision maker has a tendency to stick with the current favorite—which, in line with Dutt and Gonzalez (2012), we term choice inertia. Choice inertia may result for a variety of reasons, such as reduced or confirmatory information search (Miller and Krosnick, 1998; Russo et al., 2006).

Given that choice inertia can be expected to reduce the recency effect, Mantonakis et al. (2009) speculated that differences in choice inertia may explain that participants with little knowledge of wines showed no recency effect (see also Miller and Krosnick, 1998). People with more knowledge about the decision domain (e.g., wine connoisseurs) may be more willing or able to critically scrutinize each newly encountered option than people with less knowledge, and thus show lower (if any) choice inertia. For instance, they may be better able to evaluate an option’s attribute quality against the background of other previously experienced options and thus to accurately identify a superior option when it is encountered (e.g., Hsee et al., 1999). Finally, Mantonakis et al. (2009) proposed that choice inertia may increase for options encountered later in a sequence, as this assumption seemed to increase the fit of the model to their data.

Note that the PCM does not attribute serial-position effects in preference to primacy and recency effects in memory, and some authors have indeed argued that memory may not play a key role. Bruine de Bruin (2005), for instance, found very similar preference patterns when participants indicated their preference (a) after each encountered option and (b) after all options had been encountered. If memory were a key driver of serial-position effects in preference, one would expect stronger effects in the latter condition. Nevertheless, the contribution of memory to serial-position effects in preference is not conclusively settled (e.g., Page and Page, 2010). A critical empirical test (yet to be conducted), for instance, would be to compare the size of the recency effect when participants are asked for their preference immediately after presentation of the options or after first having worked on a distractor task (inserted after the presentation of the last option). A reduced recency effect in the latter case would indicate a contribution of memory.

Due to its simplicity, the PCM offers an elegant account of how people construct preferences among sequentially encountered options. Moreover, it poses only moderate demands on working memory and is computationally simple, making it a psychologically plausible model of preference construction (see Simon, 1955; Gigerenzer et al., 2011). One limitation, however, is that Mantonakis et al. (2009) explored the model only under the condition in which all options were of equal quality. Given that quality differences among options are common in real-life settings, however, it is interesting to examine whether the PCM also predicts serial-position effects when the quality of the options varies. In fact, it may well be that the predicted effects are strongly attenuated when there is variability in quality, as the differences in quality also shape people’s preferences. Further, it is currently unclear to what extent other potentially moderating variables (e.g., the strength of or increase in choice inertia and the sequence length) impact the PCM’s predictions of serial-position effects. Next, we set out our formal implementation of the PCM and then report the results of a computer simulation submitting the model to a systematic sensitivity analysis by varying key parameters of the decision context and the decision maker.

## METHOD

In the computer simulation, we implemented the PCM such that *L* options are sequentially encountered and each new option in the sequence is compared with the current favorite. Per default, the first option in the sequence is chosen as the initial favorite. Choice inertia was implemented as a parameter representing the probability π (0 ≤ π ≤ 1) that the current favorite is preferred over the new option irrespective of its quality. With probability 1–π, the current favorite’s quality is compared with that of the new option; at this stage, the probability that the new option is chosen as the new favorite follows from the options’ qualities. Acknowledging the probabilistic nature of choice (Mosteller and Nogee, 1951), we used Luce’s (1959) choice rule, arguably the most prominent probabilistic choice rule in decision science, to derive that probability (for alternative approaches, see Rieskamp et al., 2006; Stott, 2006). Specifically, the probability is a function of the new option’s quality, Q_new_, relative to the sum of both options’ qualities (i.e., Q_new_ + Q_old_). Overall, the probability that the new option becomes the new favorite is therefore determined as follows:

(1) $$p\left(new\right)=\left(1-\pi \right)\times \frac{Q_{new}}{Q_{new}+Q_{old}}$$

The old favorite remains with probability 1–*p*(new). The inertia parameter π was varied on four levels, π = {0;0.3;0.6;0.9}.

Quality variability was implemented as a normal distribution of *Q* (truncated between 0 and 10) with a mean of *M* = 5 and a standard deviation of δ = 5. In the conditions without quality variability, δ was set to 0. At each run of the simulation, *L* options were drawn randomly from the respective distribution and distributed randomly across the *L* positions in the sequence. We tested the PCM’s predictions at six sequence lengths: *L* = {2; 4; 6; 10; 12; 20}. (Although choice sets of 20 options may be rare in regular stores, they are common in online shopping.)

Mantonakis et al. (2009) proposed that choice inertia may increase over the sequence. We formalized this notion of dynamic choice inertia such that inertia at position *i*, π_i_, is defined as (see Yechiam and Busemeyer, 2005):

(2) $$\pi_{i}=1-\frac{(1-\pi_{1})\times 2}{1+i^{c}}$$

where π_1_ is the level of choice inertia at the first position in the sequence. The parameter *c* (0 ≤ *c* ≤ 1) governs the degree to which choice inertia increases over the positions in the sequence, with higher values indicating a stronger increase. In our simulations, we set the *c* parameter to either *c* = 0 (static choice inertia) or *c* = 0.3 (dynamic choice inertia), implementing a moderate increase of π.

For each of the 2 (quality variability) × 6 (sequence length) × 4 (choice inertia) × 2 (static/dynamic choice inertia) = 96 conditions, the model determined the favorite option at the end of the sequence. There were 100,000 runs per condition. For each condition, we determined the probability (across all runs) that an option presented at a given position in the sequence (of length *L*) would emerge as the final favorite.

## RESULTS

**FIGURE 1:**
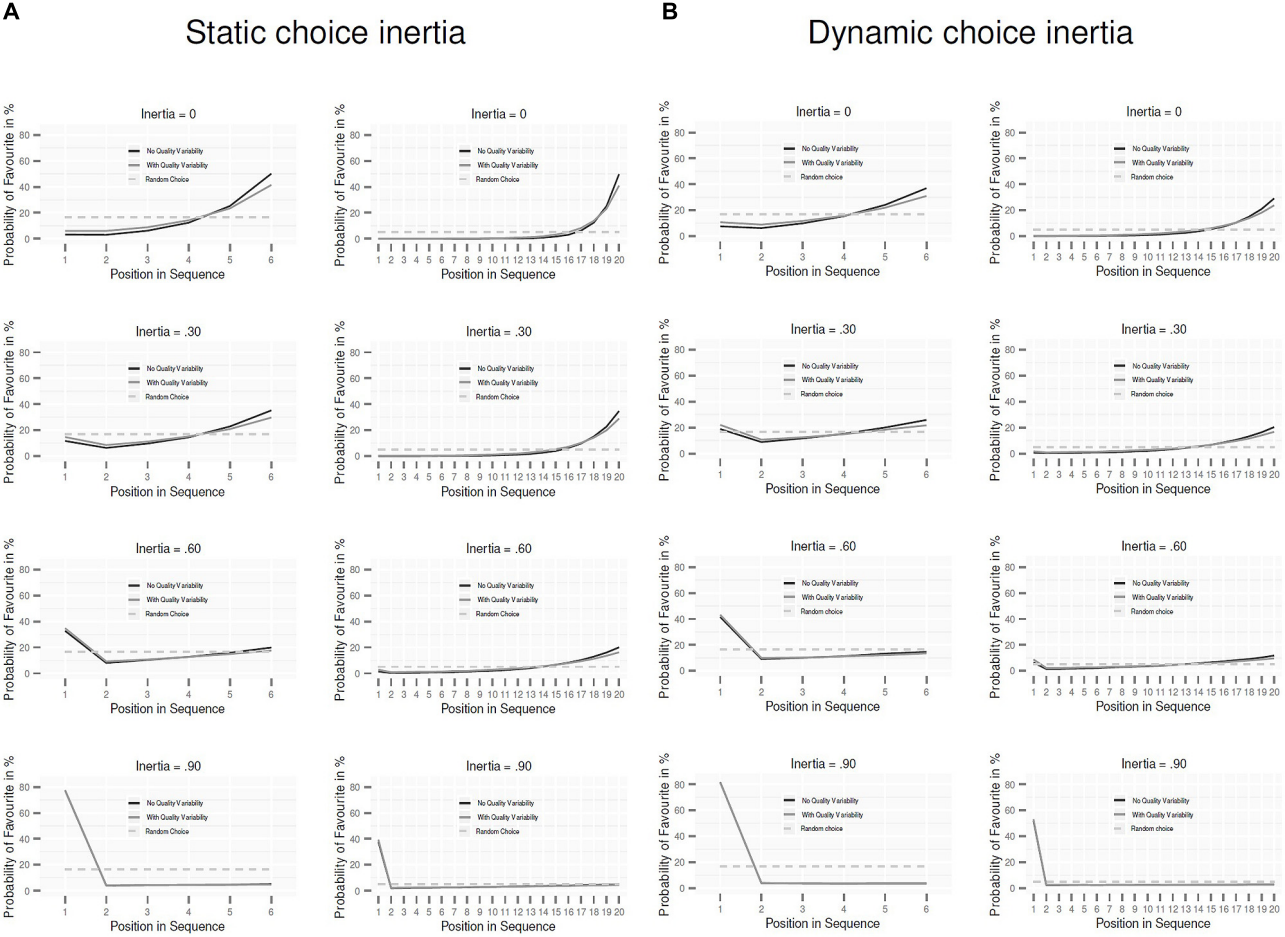
**Predictions for the pairwise-competition model with static choice inertia (A) and with dynamic choice inertia (B).** Probability that an option is the final favorite (i.e., is preferred at the end of the sequence) as a function of its position in the sequence. The gray line plots the predictions when the options differ in objective quality; the black line plots the predictions when there is no quality variability among the options. The dashed line shows the pattern under random choice.

We first focus on the conditions assuming static choice inertia. Does variability in the quality of the options affect the degree to which the PCM predicts recency and primacy effects? **Figure 1A** depicts the probability that an option is chosen as the final favorite as a function of its position in the sequence; to illustrate the impact of sequence length, the results are shown for sequence length of 6 (panels in the left column) and 20 (panels in the right column). The black line represents the preference pattern without quality variability; the gray line represents the preference pattern with quality variability. As a reference, the dashed line represents the preference pattern under random choice (i.e., where each option in the sequence has the same probability of becoming the final favorite). As can be seen, the recency effect is substantially smaller when there is quality variability than when not. The reason for this result is that, with quality variability, the probability that the object with the highest quality has already been sampled increases at later positions in the sequence. As a consequence, the probability of a new object becoming favorite decreases. Further, the difference between the preference patterns with and without quality variability diminishes at higher levels of inertia. This is due to the fact that at higher levels of inertia, quality differences play an ever weaker role.

**Figure 1A** shows that also the occurrence of a primacy effect is affected by quality variability; it is more pronounced when there is quality variability than when not. As for the recency effect, the difference between the preference patterns with and without quality variability decreases at higher levels of choice inertia. **Figure 1A** also reveals that the primacy effect emerges only if there is a substantial amount of choice inertia. This holds regardless of the existence of quality variability; without choice inertia (i.e., π = 0), there is no primacy effect at all. These findings clarify that it is choice inertia—rather than the PCM’s “first-is-best” bias (Mantonakis et al., 2009)—that is responsible for the model’s prediction of a primacy effect. The two columns of **Figure 1A** show that these general patterns hold irrespective of sequence length.

**FIGURE 2:**
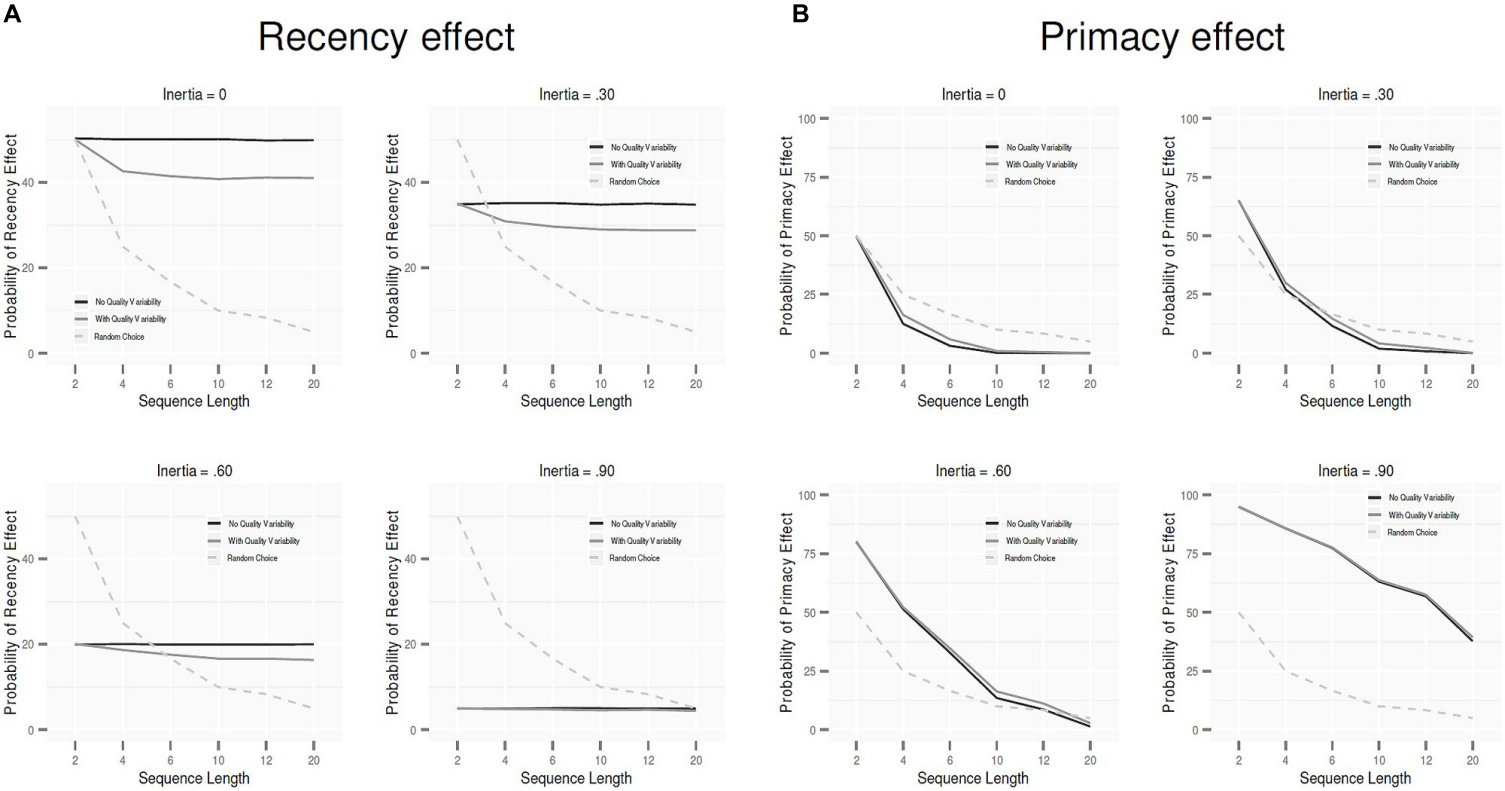
**The probability that the last option in the sequence is the final favorite (recency effect; A) and the probability that the first option in the sequence is the final favorite (primacy effect; **B**) as a function of sequence length.** The black line plots the predictions when the options do not differ in objective quality; the gray line plots the predictions when there is quality variability among the options. The dashed line shows the pattern under random choice.

How is the occurrence of primacy and recency effects affected by sequence length? **Figures 2A,B** show the probability of the effects for the different levels of *L*, again with the gray and black lines representing the pattern with and without quality variability, respectively. The dashed line represents the probability under random choice (the line shows a decrease because the probability of any option randomly becoming favorite decreases with increasing *L*). As can be seen, relative to random choice, the PCM predicts the recency effect to increase and the primacy effect to decrease with longer sequences. This holds both with and without quality variability, though the effects (relative to the pattern under random choice) are smaller with quality variability than without.

Finally, **Figure 1B** (analogous to **Figure 1A**) contrasts the predicted preference pattern with and without quality variability for the conditions with dynamic choice inertia. As can be seen, the impact of quality variability (i.e., decrease in recency and increase in primacy effects) also manifests under this condition; likewise, the impact of sequence length and level of choice inertia play out as under static inertia. Nevertheless, dynamic choice inertia does influence the choice pattern relative to static inertia. A comparison of **Figure 1B** with **Figure 1A** reveals that dynamic choice inertia decreases recency effects and increases primacy effects.

## DISCUSSION

The aim of our analysis was to examine, based on a formal implementation of the PCM, the extent to which the model’s predictions of primacy and recency effects in preference are affected by various features of the decision context—such as quality variability (an important realistic condition that was not considered in Mantonakis et al., 2009) and sequence length—and of the decision maker (e.g., choice inertia).

Our results showed that although the model predicts primacy and recency effects also under conditions of quality variability, the size of the predicted effects is clearly affected. Specifically, recency effects are reduced and primacy effects are increased when the options vary in quality. Further, choice inertia substantially reduces recency effects and increases primacy effects, and recency effects disappear altogether at high levels of inertia. In fact, our results highlight that choice inertia is the crucial variable driving primacy effects in the PCM: without choice inertia, the model does not predict primacy effects to occur (**Figure 1A**). With longer sequences the recency effect is increased, but less so with quality variability than without. The impact of quality variability also holds under dynamic, increasing choice inertia, though primacy effects are more pronounced and recency effects are weaker.

As mentioned earlier, different levels of choice inertia may be related to different levels of knowledge—a key issue in Mantonakis et al.’s (2009) investigation. What are the implications of our results for predicted differences in serial-position effects between people with high versus low knowledge? Based on **Figure 1**, decision makers with high knowledge (and low inertia) should display mainly recency effects, and decision makers with low knowledge (and high inertia), mainly primacy effects. Moreover, these differences between decision makers with low and high inertia are predicted to be more pronounced without quality variability than with.

The findings of our analysis highlight that to test the descriptive validity of the model and identify ways in which it may require further development, future studies should specifically test the predicted sensitivity of recency and primacy effects to differences in quality variability, choice inertia, and sequence length. Evidence from several existing studies seems to be consistent with the model’s predictions as elaborated in our simulations. For instance, Miller and Krosnick (1998) found larger primacy than recency effects in election competitions when less information was available and for respondents who were less knowledgeable about the candidates (see also Mantonakis et al., 2009). If lower levels of knowledge are associated with higher choice inertia, this pattern is predicted by our analyses.

The results of Bruine de Bruin’s (2005) study on voting in song competitions are also consistent with the predictions following from our model analysis. She studied experts (for whom choice inertia should be low) who were presented with a long sequence of options that (presumably) varied in objective quality. Under these conditions, the PCM predicts strong recency effects and no primacy effects. Indeed, Bruine de Bruin (2005) found sizable recency effects but no primacy effect (see also Page and Page, 2010). Overall, in addition to generating novel predictions, our results thus offer a starting point for understanding more systematically varied patterns of serial-position effects reported in the literature.

## Conflict of Interest Statement

The authors declare that the research was conducted in the absence of any commercial or financial relationships that could be construed as a potential conflict of interest.
